# Clinical features and risk factors for irritable bowel syndrome in Migraine patients

**DOI:** 10.12669/pjms.333.12379

**Published:** 2017

**Authors:** Chunlin Li, Shengyuan Yu, Huiying Li, Jin Zhou, Jieqiong Liu, Wenjing Tang, Lei Zhang

**Affiliations:** 1 Chunlin Li, MD. Department of Neurology, Chinese PLA General Hospital, Beijing 100853, China; 2 Shengyuan Yu, MD, PhD. Department of Neurology, Chinese PLA General Hospital, Beijing 100853, China; 3 Huiying Li, MS. Department of Neurology, 316^th^ Hospital of PLA, Beijing 100093, China; 4 Jin Zhou, MS. Department of Neurology, 316^th^ Hospital of PLA, Beijing 100093, China; 5 Jieqiong Liu, MD. Department of Neurology, Chinese PLA General Hospital, Beijing 100853, China; 6 Wenjing Tang, MD. Department of Neurology, Chinese PLA General Hospital, Beijing 100853, China; 7 Lei Zhang, MD. Department of Neurology, Rocket Army General Hospital, Beijing 100088, China

**Keywords:** Brain-gut axis, Central sensitization, Irritable bowel syndrome, Migraine

## Abstract

**Objective:**

Clinical and basic research increasingly suggests a correlation between migraine and irritable bowel syndrome (IBS). In this study, we aimed to explore the clinical features and risk factors for IBS in migraine patients.

**Methods:**

This was a retrospective, cross-sectional study. A total of 1,112 consecutive patients from the internal medicine and emergency departments of three hospitals from June 2014 through 2016. A comprehensive interviewer-administered questionnaire was designed based on the International Classification of Headache Disorders, 3rd edition (beta version).

**Results:**

The response rate was 94.6%. Among 1,052 participants, 287 suffered from migraine (27.3%) and 312 suffered from IBS (29.7%). A total of 79 patients suffered from both migraine and IBS (comorbidity rate: 7.5%). The migraine cohort exhibited a higher frequency of IBS than did the comparison cohort at baseline (P<0.05). Migraine patients with higher headache frequency, longer length of headache history, and anxiety disorders were more likely to also suffer from IBS (P=0.015). There were no significant differences between the two groups in age, sex, family history, duration of headache attack, migraine aura, headache intensity, or depression disorders (P>0.05). Multiple regression analysis indicated length of headache history and headache frequency were associated with IBS.

**Conclusion:**

Migraine patients with a long headache history, recurrent episodic headache attacks, and anxiety were more likely to have IBS.

## INTRODUCTION

Migraine and irritable bowel syndrome (IBS) are both functional disorders characterized by recurrent pain in the absence of detectable causes for symptoms. These seemingly distinct clinical disorders share many similarities in epidemiology, female predominance, comorbidities, and proposed pathological and physiological mechanisms. IBS is a chronic, relapsing gastrointestinal condition characterized by a set of symptoms associated with abnormal bowel function and sensation such as recurrent episodes of abdominal pain and changes in bowel habits. Similar to migraine, IBS is more common in young and middle-aged people under the age of 50 years and more predominant in women.^1^ The diagnosis of IBS is most frequently established by satisfying the symptom-based Rome 3 criteria, which include symptoms such as constipation/diarrhea, distention, abdominal pain and discomfort; in addition, organic gastrointestinal disease must be excluded. The etiologies and pathogenesis of IBS are not clear. The etiologies are related to many factors, including genetics, food inflammation, and psychological factors, among others. The pathogenesis is primarily focused on the brain-gut axis, brain gut peptides, altered gut motility, immune system, visceral hypersensitivity, etc.^2^

Migraine is defined as a common primary headache and is the sixth most common disabling disease. Clinical manifestation of migraine is a paroxysmal moderate to severe headache that seriously affects the quality of life and the labor capacity of patients.^3^ A recent national epidemiological survey estimated that the frequency of migraine in mainland China was 9.3% (5.9% for men and 12.8% for women).^4^ Clinical and basic research increasingly suggests a correlation between migraine and IBS.^5^,^6^

Although an association between IBS and migraine has been identified, the clinical features of migraine patients with IBS are rarely reported in literature. This study aimed to explore the intrinsic link between IBS and migraine and reveal the clinical features of migraine patients with IBS. This information will help provide reasonable management and preventive methods for the comorbidity.

## METHODS

This study was a retrospective cross-sectional observational study. It was conducted at the General Hospital of PLA, Rocket Army General Hospital, and the 316th Hospital of PLA from June 2014 through July 2016. Data were gathered from 1112 consecutive patients in the internal medicine and emergency departments. Only adult patients (greater than 18 years of age) were enrolled in the study. The diagnosis of IBS was based on the Rome III criteria.^7^ The migraine diagnostic criteria were based on the International Classification of Headache Disorders, 3rd edition (beta version) (ICHD-3-beta).^8^ All 1112 patients were asked to complete a series of questionnaires consisting of validated components on IBS symptomatology and migraine symptomatology. IBS was diagnosed according to the Rome 3 Criteria: Recurrent abdominal discomfort or pain, 3 days per month in the last 3 months (12 weeks); associated with ≥2 of the criteria below. The criteria were fulfilled with the following symptoms for six months before diagnosis.

1) Improvement with defecation;

2) onset associated with a change in stool frequency;

3) onset associated with a change in stool form (appearance).

The headache questionnaires included general state (including sex, age, anamnesis, etc.) and headache characteristics (including duration, frequency, intensity, inducement, etc.). Headache frequency was assessed by the average number of days with headache per month. Headache pain severity was assessed using the visual analogue score (VAS), which ranges from 0 to 10. Complications such as anxiety and depression disorders were assessed by the Self-Rating Anxiety Scale (SAS) and Self-Rating Depression Scale (SDS), respectively.

Statistical analyses were performed using SPSS Statistics version 16.0. Normally distributed data that obeyed the normal distribution is expressed as means ± standard deviations; enumeration data is expressed as percentages. For single factor analyses, the data were analyzed by variances, and the LSD-t method was used to compare groups. Enumeration data was assessed using the Kruskal-Wallis H test. IBS was the dependent variable in the multivariate analysis (logistic stepwise regression analysis) to determine whether clinical features of migraine were risk factors for IBS.

### Ethical Considerations

All participants provided informed consent. This study was approved by the Institutional Review Board of the Chinese PLA General Hospital and the Military Medical Postgraduate College.

## RESULTS

Among 1,110 patients, 48 submitted incomplete or unreliable questionnaires, and 1,052 completed the survey. The response rate was 94.8%. The median age of respondents was 41.5 years and 706 respondents were female. Among 1,052 participants, 287 suffered from migraine (27.3%) and 312 suffered from IBS (29.7%); 79 patients suffered from both migraine and IBS (comorbidity rate: 7.5%).

### Baseline demographic status and comorbidity in migraine and comparison cohorts

A comparison group of 287 participants without migraine was matched to the migraine group according to sex and age. The demographics and comorbidities of the migraine and comparison cohorts are shown in Table-I. Both groups were similar in average age and smoking and drinking habits. The migraine cohort had a higher frequency of IBS than did the comparison cohort at baseline (P < 0.05).

**Table-I:** Baseline data for comparison and migraine cohorts.

	*Migraine (n = 287)*	*No migraine (n = 287)*	*P value*	*Odds ratio (95% CI)*
Age (yr)	41.3 ± 8.2	40.9 ± 7.1	0.53	0.86~1.66
Women	195 (67.9)	195 (67.9)	--	--
Smoking	35 (12.2)	42 (14.6)	0.26	0.50~1.31
Alcohol	55 (19.2)	48 (16.7)	0.51	0.77~1.81
Body mass index (kg/m^2^)*	18.5 ± 7.5	16.7 ± 7.8	0.005	0.55~3.05
IBS*	79 (27.5)	48 (16.7)	0.003	1.27~1.82
Anxiety*	85 (29.6)	52 (18.1)	0.002	0.80~1.59
Depression*	32 (11.1)	15 (5.2)	0.01	1.22~4.24

Values are expressed as mean ± SD or n (%).

* P <0.05 between the migraine group and no migraine group.

### Comparison of clinical features between migraine patients with and without IBS

We divided migraine patients into two groups based whether they also had IBS and compared the clinical characteristics of each group. We found headache frequency, length of headache history, and presence of anxiety disorders were closely correlated with IBS (P values of < 0.001, 0.004, and <0.001, respectively). There were no significant differences between the two groups in age, sex, family history, headache attack duration, migraine aura, headache intensity, or presence of depression disorders (P>0.05 for all). In this study the ratio of male to female migraine patients was approximately 1:4, but there was not a significant difference in the male: female ratio between the groups with and without IBS. Table-II.

**Table-II:** Clinical features in migraine patients with and without IBS.

	*With IBS (n = 79)*	*Without IBS (n = 208)*	*P value*	*Odds ratio (95% CI)*
Age (yr)	40.2 ± 8.0	39.7 ± 7.9	0.58	0.39~1.60
Women	47 (59.5)	148 (71.2)	0.11	0.34~1.12
Family history of migraine	25 (31.6)	70 (33.7)	0.61	0.48~1.45
Headache frequency				
<7 d/mon	39 (49.4)	155 (74.5)		
≥7d/mon	40 (50.6)	53 (25.5)	0.0001	0.20~0.57
Headache attack duration (hrs)	16.5 ± 7.5	16.7 ± 7.8	0.78	-1.71~2.27
Migraine aura	12 (15.2)	26 (12.5)	0.55	0.60~2.62
Headache intensity (0-10 points)	7.0 ± 2.1	7.1 ± 2.2	0.60	-0.41~0.71
Length of headache history (yrs)	13.7 ± 7.1	11.1 ± 5.9	0.004	-4.43~-0.87
Anxiety	43 (54.4)	62 (29.8)	<0.001	2.24~6.77
Depression	8 (10.1)	18 (8.6)	0.70	0.50~2.85

Values are expressed as mean ± SD or n (%).

### Clinical characteristics of migraine and correlation with IBS

In the multifactor logistic regression analysis, IBS was the dependent variable, and age, sex, family history, length of headache history, headache attack duration, migraine aura, headache intensity, headache frequency, and presence of anxiety and depression disorders were the independent variables. Multivariate analysis revealed a higher risk of IBS in migraine patients with longer headache history OR=1.07 (95% CI, 1.02-1.12), P=0.003. And higher frequency of headache attacks OR=2.93 (95% CI, 1.22 -7.07), P=0.02 (Table-III).

**Table-III:** Logistic stepwise regression analysis of migraine patients for factors influencing IBS risk.

*Variables*	*SE*	*u value*	*P value*	*OR*	*95%CI*
Length of headache history	0.02	3.00	0.003	1.07	1.02~1.12
Headache attack duration	0.02	0.07	0.95	1.00	0.97~1.04
Family history	0.39	1.26	0.21	1.63	0.77~3.46
Anxiety	0.44	1.20	0.23	1.70	0.71~4.04
Age	0.02	0.29	0.77	1.01	0.97~1.04
Headache frequency	0.45	2.39	0.017	2.93	1.22~7.07
Headache intensity	0.07	0.86	0.39	0.94	0.83~1.08
Migraine aura	0.50	0.09	0.94	1.04	0.39~2.7
Sex	0.35	1.96	0.05	0.50	0.25~1.00
Depression	0.53	1.60	0.11	0.43	0.15~1.21

## DISCUSSION

IBS and migraine are both common functional disorders characterized by recurrent pain in the absence of detectable causes. The reported frequency of IBS ranges from 5 to 20%.^9^ Migraine similarly affects 10%–12% of the adult population.^10^ Recent studies suggest that migraine may be associated with IBS.^5^,^11^ A study of 125,000 patients with IBS found that the frequency of migraine in IBS patients was three times greater that of normal subjects;^5^ the incidence of IBS in migraine patients was also significantly increased.^11^ In our study, the incidence of IBS in migraine patients was significantly higher than that in the group without migraine (27.5% vs. 16.7%, P<0.05). These findings are similar to those of the previous studies, and further support the view that migraine is closely related to IBS.

Which migraine patients are more likely to have IBS as comorbidity? In our study, single factor analysis showed that the susceptibility to IBS in migraine patients might be related to headache frequency, length of headache history, and presence of anxiety disorder. Similarly, multivariate analysis revealed a higher risk of IBS in migraine patients with longer headache history and higher headache attack frequency. We observed a close correlation of headache frequency and length of headache history with IBS in our study participants. Similarly a higher risk of IBS in migraine patients with longer headache history and higher frequency of headache attack was observed in our study. These data provide robust evidence of links between chronic and recurrent migraine attacks and IBS, a chronic relapsing gastrointestinal condition. The central sensitization theory may explain these links. Recurrent migraine attacks lead to sensitization of the pain receptor of the trigeminal nerve on meninx, resulting in central sensitization of the caudate nucleus of the trigeminal nerve. Central sensitization induces changes in sensory input process conduction to the brainstem, especially surrounding the caudate nucleus of trigeminal nerve and periaqueductal gray matter, leading to chronic paroxysmal migraine.^12^,^13^

Meanwhile, there is a clinically close relationship between gastrointestinal motility disorders and intestinal neuronal abnormalities. Activation of the brain-gut axis network may be one pathophysiological mechanism of the comorbidity between migraine and IBS.^14^,^15^ The brain-gut-axis is a bidirectional regulation axis between the central nervous system (CNS) and gastrointestinal tract functioning. It is a neuro-endocrine network composed of the CNS, peripheral nervous system, autonomic nervous system, enteric nervous system (ENS).^16^ Information obtained by peripheral nervous system, autonomic nervous system and ENS are continuously integrated and processed by the CNS.^17^ For example, when the autonomic nervous system receives repeated stimulation, the body would be in a sensitive state of defense. The ENS and the brain-gut-axis network in IBS patients would be activated through psychological and immune pathways, altering 5-HT concentration.^18^ 5-HT is an important neurotransmitter for the regulation of gastrointestinal tract functioning and has also been implicated in the pathogenesis of migraine. Apart from the brain-gut-axis theory, anatomical evidence exists for vulnerability of migraine patients to IBS. Migraine is often accompanied by nausea, vomiting, and other autonomic symptoms, suggesting excitement of the vagus nerve in migraine patients. The vagus nerve is the longest brain nerve and has the widest distribution. Efferent vagal fiber terminals form synaptic contacts with postganglionic neurons, primarily in the myenteric nervous plexus, allowing a variety of information collected from intestinal tract to be transmitted to the brain and connecting paroxysmal migraine and gastrointestinal symptoms.^19^

The ENS can sense, initiate, and regulate the movement and secreting function of the gastrointestinal tract. Similar to the pathophysiological mechanisms of migraine, IBS recurrently activates the intestinal nervous system, leading to C-FOS expression in the brain stem and causing central sensitization.^20^,^21^

In our study, participants mood disorders were also investigated. Single factor analysis indicated a high rate of anxiety disorder in migraine patients with IBS compared with those without IBS (P<0.001). The visceral sensory information and mood processing systems influence one another in the limbic system, allowing mental stress to enhance visceral sensitivity and explaining why functional gastrointestinal diseases often accompany mood disorders. The vagus nerve can regulate the expression of neurotransmitters and transmit signal molecules released by intestinal flora to the brain to regulate brain function and influence the anxiety- and depression-like behavior of the host.^22^ Thus, the treatment of IBS aims to control not only intestinal symptoms, but also accompanying mood disorders. A number of clinical and epidemiological studies have confirmed migraine patients often have depression and anxiety disorders,^23^ and patients with these disorders have a higher risk of migraine.^24^ Clinically, the tricyclic anti-anxiety and anti-depressant drug amitriptyline alleviates the symptoms of both migraine and IBS, suggesting that migraine, IBS, and anxiety/depression disorders may share a common etiology. However, there was no statistically significant difference in depression between groups in this study; this may be due to the small research sample size and low morbidity of depression in these subjects.

To the best of our knowledge, this is the first study to detect the association between IBS and clinical features of migraine. However, our study has some limitations. First, 27.5% of the migraine patients in our study suffered from IBS, but there is a potential bias in that patients with more frequent medical-care-seeking behavior may have a higher chance of being diagnosed with IBS and migraine compared with the general population. Second, the development of chronic migraine is associated with other risk factors such as sleep apnea/snoring, obesity, painkiller use, body mass index, physical activity, socioeconomic status, etc. that may contribute to the constipation/diarrhea manifestations of IBS. However, data on these factors were not collected in this study. Further randomized large-scale studies on the link between migraine and irritable bowel are necessary to provide better control and treatment methods for the migraine/IBS comorbidity.

### Conclusion

The frequency of IBS in migraine patients was 27.5%. The migraine patients with long headache history, high headache frequency, and anxiety were more prone to be affected with IBS. The comorbidity of migraine and IBS may be attributed to the brain-gut axis and central sensitization. However, in-depth studies are still in need to clarify the underlying mechanism.

### Authors` Contribution

**CL** conducted the survey, analyzed the raw data, performed statistical analyses, interpreted the data, drafted the manuscript, and prepared the manuscript for submission. **HL & JZ** conducted the survey. **SY** interpreted the data and reviewed the manuscript. **WT & LZ** reviewed the manuscript. All authors participated in the planning of the study and approved the final manuscript for submission.
